# The Degradation and Repolymerization Analysis on Solvolysis Liquefaction of Corn Stalk

**DOI:** 10.3390/polym12102337

**Published:** 2020-10-13

**Authors:** Weisheng Chen, Qinqin Zhang, Xiaoqi Lin, Kaisen Jiang, Dezhi Han

**Affiliations:** 1 Shandong Provincial Key Laboratory of Biochemical Engineering, College of Marine Science and Biological Engineering, Qingdao University of Science and Technology, Qingdao 266042, China; chenweisheng0223@163.com (W.C.); qqzhang@qust.edu.cn (Q.Z.); lxq15621496181@163.com (X.L.); j15192848709@163.com (K.J.); 2 State Key Laboratory Base of Eco-chemical Engineering, College of Chemical Engineering, Qingdao University of Science and Technology, Qingdao 266042, China

**Keywords:** lignocellulosic biomass, liquefaction, corn stalk, residue, repolymerization, utilization

## Abstract

One of the most effective and renewable utilization methods for lignocellulosic feedstocks is the transformation from solid materials to liquid products. In this work, corn stalk (CS) was liquified with polyethylene glycol 400 (PEG400) and glycerol as the liquefaction solvents, and sulfuric acid as the catalyst. The liquefaction conditions were optimized with the liquefaction yield of 95.39% at the reaction conditions of 150 °C and 120 min. The properties of CS and liquefaction residues (LRs) were characterized using ATR–FTIR, TG, elemental analysis and SEM. The chemical components of liquefied product (LP) were also characterized by GC–MS. The results indicated that the depolymerization and repolymerization reaction took place simultaneously in the liquefaction process. The depolymerization of CS mainly occurred at the temperature of <150 °C, and the repolymerization of biomass derivatives dominated at a higher temperature of 170 °C by the lignin derivatives repolymerization with cellulose derivatives, hemicellulose derivatives and PEG400 and self-condensation of lignin derivatives. The solvolysis liquefaction of CS could be classified into the mechanism of electrophilic substitution reaction attacked by the hydrogen cation.

## 1. Introduction

The abundant and valuable lignocellulosic biomass could be considered as an alternative to supplement or even replace fossil resources for the synthesis of various chemicals. Lignocellulosic biomass consists of three major components (cellulose, hemicellulose and lignin) with plenty of functional groups, such as hydroxyl and phenolic hydroxyl, methoxyl and carboxyl, etc. [1]. Due to the potential advantages of improving the functionality of the derived biopolymers and benefiting the economic and environmental aspects, the lignocellulosic biomass could be used as raw material for producing the biomass-based chemicals [2,3].

The lignocellulosic biomass can be physically, thermochemically or biologically transformed into intermediate chemicals. Typically, the thermochemical conversion process can be effectively implemented via gasification, liquefaction and carbonization. Although biomass gasification for power generation has been industrially applied, the treatment and recycling of wastewater for preventing secondary pollution still need to be further optimized. Through carbonization with postmodification, the biomass can be turned into carbon fiber, solid acid catalysts and activated carbon, etc. [4,5]. Presently, the liquefaction has been demonstrated as the most effective method for transforming lignocellulosic biomass into high value-added intermediates [6], such as bio-polyol and bio-crude oil [7,8,9]. During the liquefaction process, the lignocellulosic biomass may be decomposed into small fragments with low molecular weight in the presence of solvents and catalysts [10]. Direct liquefaction is the extraction process of biomass with the assistance of a supercritical or subcritical fluid. However, the high temperature (250–400 °C) and high pressure (5–40 MPa) are usually required for the solvent to reach a supercritical state during the direct liquefaction process [11], which would limit its application in the practical production [12]. Compared to direct liquefaction, solvolysis liquefaction, with the merits of low reaction pressure (1 atm) and temperature (100–200 °C), is a more preferable method, which can convert lignocellulosic biomass into valuable intermediates under relatively mild reaction conditions [13,14,15]. Moreover, the selection of solvents for the solvolysis liquefaction is very important to prevent liquefied products from cross-linking and repolymerizing [16]. Polyethylene glycol (PEG) is one of the effective liquefaction solvents for solvolysis liquefaction of lignocellulosic biomass [17]. In order to achieve high liquefaction yield, co-solvents, such as PEG-glycerol and PEG-ethylene glycol, have been widely used in the solvolysis liquefaction system [18,19]. Comparing with the single solvent of PEG, the addition of glycerol could decrease the residue content due to its resistance to the recondensation of liquefied products [19]. Subsequently, some assisted technologies, such as liquid-phase microplasma or microwave–ultrasonic-assisted technology, have also been employed to improve the liquefaction yield [20,21]. Furthermore, several new types of catalysts, such as mesoporous catalysts [22], cation exchange resin [23] and ionic liquids [24], were also applied to the solvolysis liquefaction. To date, sulfuric acid is still regarded as the preferred catalyst because of its superior catalytic performance, with the liquefaction yield of >95% [24]. As summarized above, most of the researchers mainly focused on the optimization of liquefaction conditions or the utilization of final liquefied products. However, the liquefaction and repolymerization processes during the solvolysis liquefaction of the lignocellulosic biomass still need further investigation.

The aim of this work was to examine the effect of liquefaction conditions on the solvolysis liquefaction process of corn stalk (CS) and determine the depolymerization and repolymerization mechanism in the liquefaction process by evaluating the characteristic groups, thermal behavior, morphology and elemental content of the CS and liquefaction residues (LRs).

## 2. Material and Methods

### 2.1. Materials

CS was collected from Rizhao, Shandong province, China. The holocellulose was isolated from CS, according to GB T 2677.10-1995. The cellulose and hemicellulose content was determined based on the method, as previously reported [25,26]. The lignin content of CS was measured according to the method from the national renewable energy laboratory [27]. The contents of cellulose, hemicellulose and lignin in CS were 35.74 wt %, 26.79 wt % and 14.00 wt %, respectively. Before liquefaction, the CS was ground into powders with a particle size of <250 μm using a universal pulverizer, followed by drying in the oven at 108 °C overnight. PEG400 and glycerol were purchased from Sinopharm Chemical Reagent Co., Ltd. (Shanghai, China). Concentrated sulfuric acid (98.3 wt %) and acetone were purchased from Yuandong Fine Chemical Co., Ltd. (Yantai, China). BSTFFA+TMCS (99:1, >98%) was supplied by Shanghai EKEAR Bio@Tech Co., Ltd. (Shanghai, China).

### 2.2. Liquefaction Process

**Figure 1:**
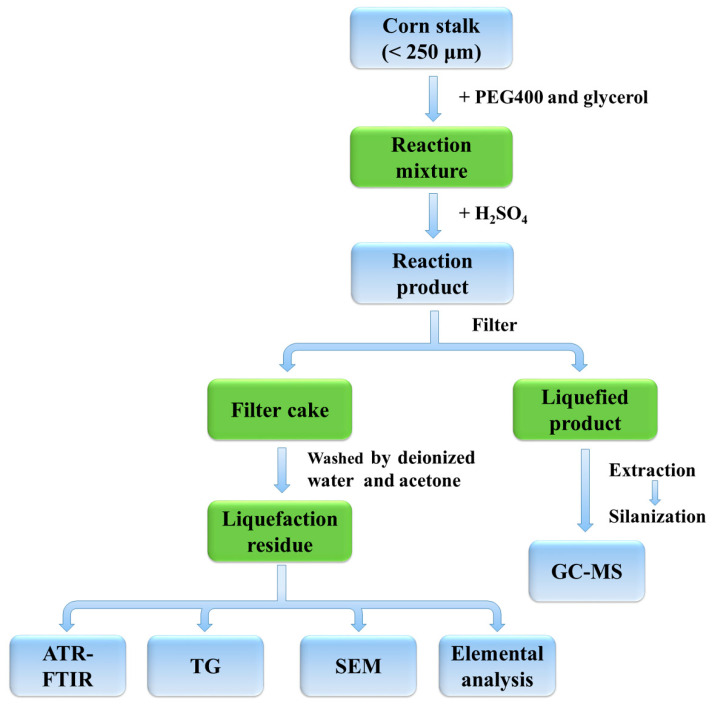
The procedures of the solvolysis liquefaction process and the subsequent characterizations.

The mixture of the preweighed CS, PEG400 and glycerol was first added into a 100 mL three-necked flask under magnetic stirring. Then the sulfuric acid serving as a catalyst was dropped into the above mixture under the temperature of 130–170 °C. Finally, the reaction product was immediately transferred into a Buchner funnel for filtration. The filter cake was washed with deionized water and acetone, respectively, until the filtrate was colorless, then dried overnight in an oven at a temperature of 108 °C. As shown in Figure 1, the obtained liquefaction residue (LR) and liquefied product (LP) were collected separately for subsequent analysis. The corresponding LRs obtained under the liquefaction temperature of 130, 150 and 170 °C were denoted as LR-130, LR-150 and LR-170, respectively. The liquefaction yield of CS can be calculated by the following formula:

(1) $$\mathrm{Liquefaction}\;\mathrm{yield}=\frac{M_{\mathrm{CS}}-M_{\mathrm{LR}}}{M_{\mathrm{CS}}}\times 100\%$$

where M_CS_ stands for the weight of the CS added to the reaction system, and M_LR_ is the weight of LR.

### 2.3. Characterization of the Corn Stalk and Liquefaction Residues

The infrared spectroscopic analysis of CS and LRs was performed on an attenuated total reflectance Fourier-transform infrared spectrometer (ATR-FTIR) of Bruker VERTEX 70 (Bruker Optik GmbH, Ettlingen, Germany) with the wavenumber range from 600 to 4000 cm^−1^ at a resolution of 2 cm^−1^. TG tests were carried out on a thermal analyzer of TGA/DSC 1SF (Mettler Toledo, Zurich, Switzerland) with an inert nitrogen atmosphere in the temperature range of 30 to 800 °C (heating rate: 10 °C·min^−1^). The analysis of elemental contents in CS and LRs were conducted on a Vario Elementar EL III element analyzer (Elementar Analysensysteme GmbH, Hanau, Germany). The higher heating value (HHV) reflected primarily by carbon (C), hydrogen (H) and oxygen (O) in the CS and LRs was calculated by the Dulong’s formula as follows [28]:

HHV (MJ kg^−1^) = 0.3383 C+1.442 (H–O/8) (2)

where C, H and O are the weight percentage of carbon, hydrogen and oxygen in the samples, respectively. Morphology of the samples was observed via a Hitachi S-4800 SEM (Hitachi High-Technologies Corp, Tokyo, Japan) with a backscattered electron detector at 3 kV.

### 2.4. Characterization of the Liquefied Product

Before the Gas Chromatography-Mass Spectrometer (GC–MS) characterization, the silanization of LP was necessary to reduce its high boiling point. First, the LP was extracted three times with methylene chloride. The extracted liquid was blow-dried with nitrogen. Then, then 80 μL pyridine and 150 μL BSTFA+TMCS were added into the extracted liquid for silanization at 70 °C for 45 min. Subsequently, the sample was filtered using a microporous filtration membrane of 0.22 μm and then used for the measurement [29]. The compositions of silanized LP were analyzed by GC–MS of 7990B-7000C (Agilent Technologies, Santa Clara, CA, USA) with HP-5 quartz capillary column (30 m × 0.25 mm × 0.25 μm).

## 3. Results and Discussion

### 3.1. The Investigation on the Liquefaction Conditions of Corn Stalk

**Figure 2:**
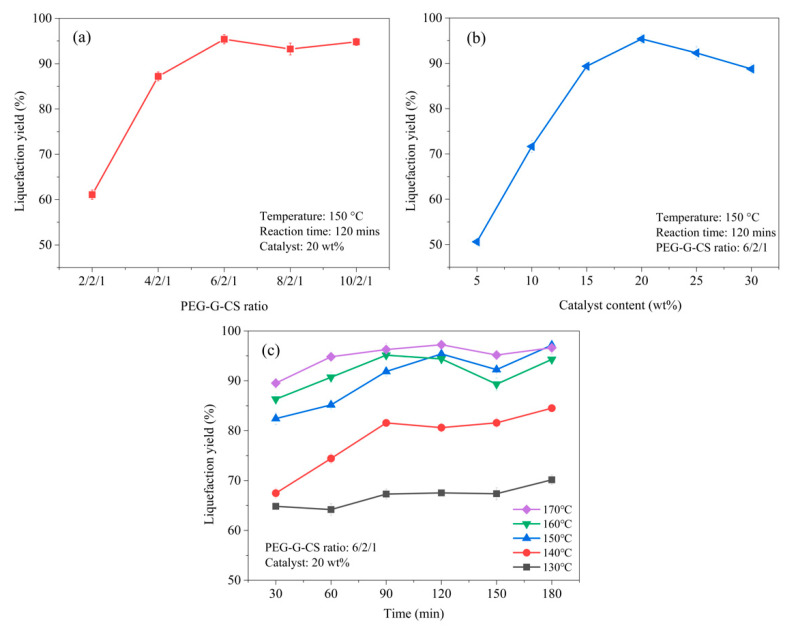
The liquefaction yield of corn stalk (CS) under different reaction conditions of the mass ratio of PEG400, glycerol and CS (PEG–G–CS ratio) (**a**), catalyst content (**b**), reaction temperature and time (**c**).

The liquefaction conditions significantly influenced the liquefaction yield during the solvolysis liquefaction process of the CS. It can be observed from Figure 2a that the liquefaction yield of CS increased from 69.73% to 89.75% as the PEG–G–CS ratio (mass ratio of PEG400, glycerol, and CS) changed from 2/2/1 to 6/2/1 and then nearly reached a plateau. At the condition of the low PEG–G–CS ratio, the CS could not mix intensively with solvents, while the sulfuric acid also could not effectively catalyze the liquefaction process, resulting in the low liquefaction yield. Furthermore, liquefaction yield increased with the increase in the catalyst content up to 20 wt % (relative mass percentage to CS), then decreased gradually, as depicted in Figure 2b. Generally, a large amount of catalyst corresponds to more catalytic active centers, which are responsible for the superior catalytic performance [30]. However, sulfuric acid with high concentration (>20 wt %) in the liquefaction mixture enhanced the complex repolymerization reaction in the solvolysis liquefaction of CS, thus resulting in the decline of liquefaction yield. Figure 2c shows the influence of liquefaction temperature and time on the liquefaction yield of CS. It can be seen that the high temperature had a positive effect on the liquefaction yield, revealing the thermal domination in the liquefaction reaction [13]. When the temperature was ≤140 °C, the glycosidic linkages in the CS decomposed into smaller soluble fragments with the reaction of dehydration and decarbonylation under the catalysis of sulfuric acid [31]. The liquefaction yield tended to be stable after the reaction time was >90 min. In the case of temperature ≥150 °C, the partial degradation of the crystalline cellulose occurred, as reported in previous literature [32]. Moreover, the liquefaction yield at each investigated temperature reached the maximum value at the reaction time of 120 min. After the comprehensive consideration of liquefaction yield and energy efficiency, the liquefaction temperature of 150 °C and the reaction time of 120 min were selected for the subsequent experiments.

### 3.2. The Analysis of Chemical Functional Groups

**Figure 3:**
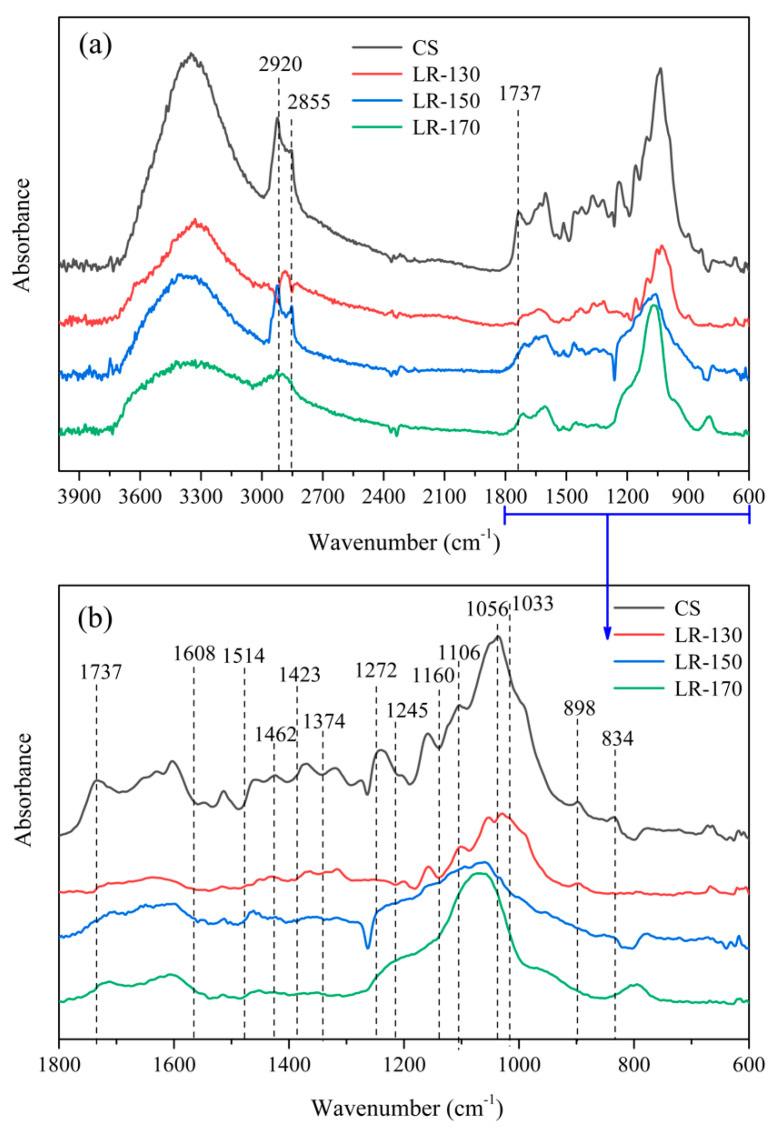
The ATR–FTIR spectra of CS and liquefaction residues (**a**), the distinctive region from 600–1800 cm^−1^ of the ATR–FTIR spectra (**b**).

Figure 3a illustrates the ATR–FTIR spectra of CS and LRs. A broad hydroxyl adsorption peak can be observed in the range of 3000–3750 cm^−1^, corresponding to the absorption bands of the alcoholic hydroxyl group in cellulose and hemicellulose as well as the phenolic hydroxyl group in lignin [33]. The sharp peaks at around 2920 cm^−1^ and 2855 cm^−1^ are ascribed to the stretching vibration of–CH_3_ and–CH_2_ in cellulose, hemicellulose and lignin, respectively [34]. The distinctive region from 600–1800 cm^−1^ is depicted in Figure 3b for investigating the intensity transformation of three main components in CS and LRs. The characteristic absorption peaks of cellulose are at 1425, 1374, 1250, 1160, 1056 and 898 cm^−1^, and peaks at 1737, 1608, 1423 and 1245 cm^−1^ are associated with hemicellulose, while the absorption bands related to the lignin are at 1514, 1462, 1272, 1106, 1032 and 834 cm^−1^ [35,36,37,38]. The relative intensity of the characteristic peaks of those three main components in LRs decreases, as compared to the parent CS, indicating the effective conversion of the CS to LP during the liquefaction process. The lignin decomposed through the dominant bonds of β–O–4, 4–O–5 and dibenzodioxocin units during the solvolysis liquefaction process [23], while the cellulose and hemicellulose were broken up through the cleavage of C–O bond [39,40]. Furthermore, when the liquefaction temperature increased from 130 to 170 °C, the intensity of absorption peak at 1000–1100 cm^−1^ decreased slightly, and then increased obviously, suggesting that these bands of the LRs came from the C–O–C vibration from both CS and PEG400. These results reveal that the depolymerization of CS and the repolymerization of biomass derivatives took place simultaneously in the liquefaction process, especially at the high liquefaction temperature of 170 °C [41]. Since the phenolic hydroxyl groups are more reactive than aliphatic hydroxyl groups, cellulose and hemicellulose derivatives are more likely to react with lignin derivatives instead of solvents, while the lignin derivatives probably reacted with PEG400 and underwent self-condensation at high temperatures to form insoluble substances into the LRs [42,43].

### 3.3. Thermal Behaviors of the Corn Stalk and Liquefaction Residues

**Figure 4:**
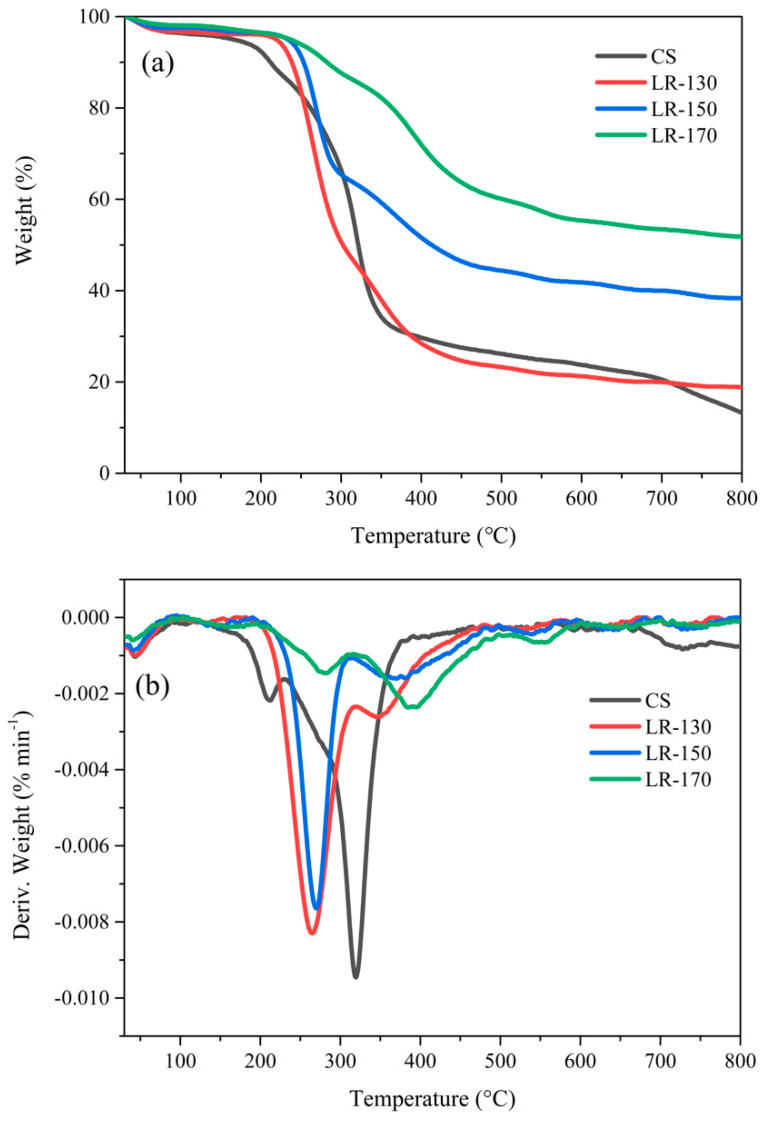
TG (**a**) and DTG (**b**) curves of CS and LRs.

Figure 4 shows the TG and DTG curves of CS and LRs for further interpretation of the solvolysis liquefaction process of CS. In the case of CS, as presented in Table 1, the weight loss in the earlier stage (<210 °C) was mainly attributed to the water evaporation and the initial pyrolysis of hemicellulose, followed by the intensive thermal degradation of the hemicellulose, cellulose and lignin in the temperature range of 210–400 °C with a noticeable DTG peak at 320 °C. However, the LRs exhibited different thermal decomposition behaviors according to the analysis of the TG and DTG results. The maximum weight loss peaks in the DTG curve of LR-130 and LR-150 shifted to low temperature (270 °C), which was equivalent to a reduction of 50 °C in comparison with the result of CS. This implies the liquefaction process partially broke down the building blocks of hemicellulose, cellulose and lignin in CS into polyols, resulting in abundant constituents with easy pyrolysis characteristics in the LRs. This phenomenon was also found in the liquefaction process of peanut shells, as reported in our previous work [23]. Moreover, when the liquefaction temperature was elevated to 170 °C, the maximum weight loss peaks in the DTG curve of LR-170 shifted to the high temperature of 392 °C, demonstrating the high thermal stability of the LR-170. This may be attributed to the more content of undecomposed lignin in the LR-170, which is consistent with the results that lignin derivatives could easily repolymerize with other derivatives and PEG400 in the liquefaction process. Moreover, the final char content of CS and LR-130, LR-150 and LR-170 was 13.2%, 18.8%, 38.5% and 52.0%, respectively, which may also reflect the high thermal stability of LR obtained under the liquefaction condition of high temperature.

In Table 1, T_Max1_, T_Max2_ and T_Max3_ represent the temperature at the first, the second and the third maximum weight-loss rate in the DTG curves, respectively.

### 3.4. Elemental Analysis of Corn Stalk and Liquefaction Residues

The elemental compositions and HHV calculations of CS and LRs are summarized in Table 2. It can be seen that the carbon content and the HHV of each LR were both lower than that of CS, while the oxygen content of each LR showed the opposite trend. The increase in oxygen content was contributed by the oxygen-enriched source of PEG400, which is considerable proof that lignin derivatives repolymerized with PEG400 in the liquefaction process [43]. Moreover, the high liquefaction temperature resulted in a decrease in the H/C ratio of the LRs. When the liquefaction temperature was elevated from 130 to 170 °C, the H/C ratio of LRs decreased from 0.1428 to 0.1167. Moreover, the HHV of the LRs also had a similar trend in comparison with the H/C ratio. The low H/C ratio and HHV of LRs, especially the LR-170 indicated the high thermal stability of the obtained LRs, which was consistent with the results of the TG and DTG analysis (Figure 4).

### 3.5. Morphology of the Corn Stalk and Liquefaction Residues

**Figure 5:**
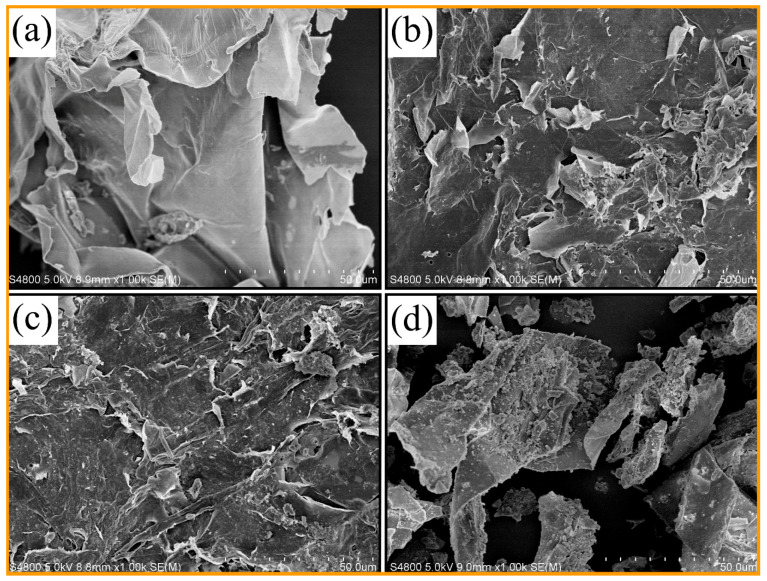
SEM images of CS (**a**), LR-130 (**b**), LR-150 (**c**) and LR-170 (**d**).

The SEM images of CS and LRs obtained under different liquefaction temperatures are shown in Figure 5. It can be observed that the original CS showed a smooth surface and intact lamellar structure. After the liquefaction reaction, a rough and fracture surface of LRs was observed, as shown in Figure 5b–d. The hemicellulose and lignin generally enwrap the microfibers, which are formed by the assembly of cellulose molecules and play a binding role in lignocellulosic biomass [44,45]. Therefore, the solvolysis liquefaction reaction primarily occurred on the surface of CS to degrade amorphous lignin and hemicellulose [6], as demonstrated by the small pores on the surface of LRs. Moreover, many small granules attached to the surface of LRs were found, especially in the LR-170. This may be attributed to the left debris after the depolymerization and reaggregation product due to the repolymerization reaction, as previously mentioned.

### 3.6. Depolymerization and Repolymerization Mechanism of Corn Stalk in the Solvolysis Liquefaction Process

The LP under the liquefaction temperature of 150 °C and reaction time of 120 min was analyzed by GC–MS. The main components can be found in Table S1 (Supporting Information). According to GC–MS analysis, the chemical components of the LP generally can be divided into five categories based on the functional groups, i.e., alkanes, acids, ethers, esters and phenolic compounds. Aromatic compounds such as LP-A (RT = 12.548) and LP-B (RT = 22.722) correspond to the degradation of lignin. The primary polyether compounds in the liquefied products arose from the polymerization of molecules, LP-C (RT = 28.894) and LP-D (RT = 34.782) in PEG400, due to the high content of solvents in the liquefied products. A few acids derived from liquefied products, such as n-hexadecanoic acid (RT = 23.86) and octadecanoic acid (RT = 25.721), were observed, and they have long chain structures with the carbon number of 14–18. There were likely a few fatty acids with long-chain structures present in CS before liquefaction [46].

**Figure 6:**
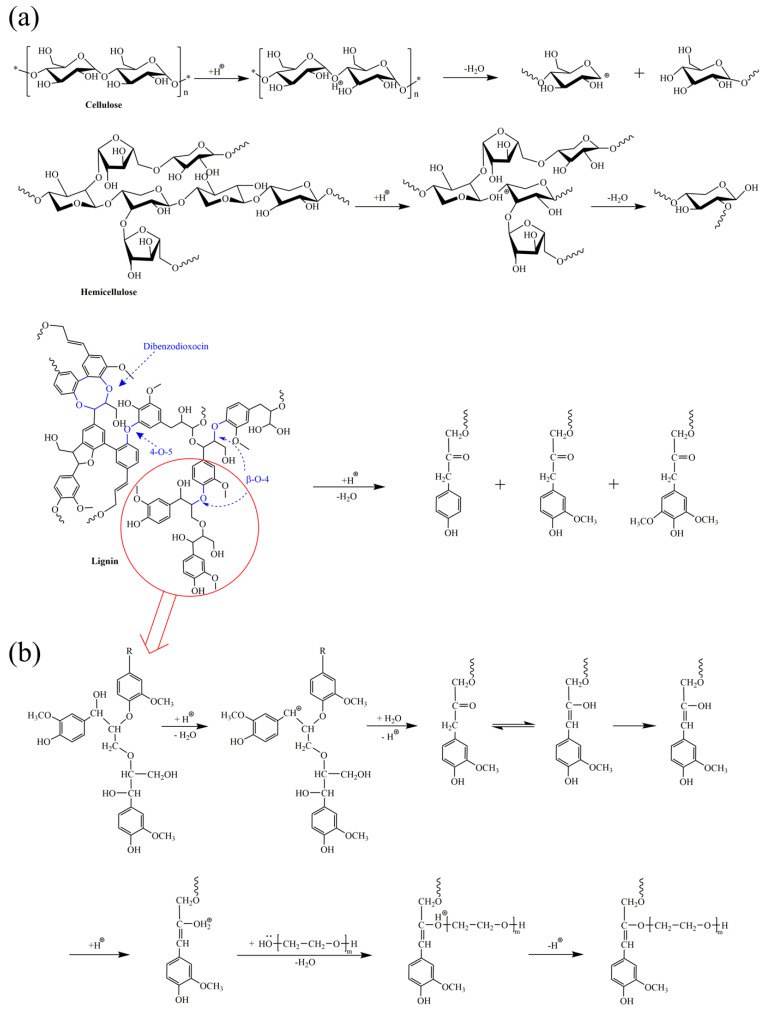
Proposed depolymerization of cellulose, hemicellulose and lignin in CS (**a**), and repolymerization pathways for lignin derivatives and PEG400 (**b**) in the solvolysis liquefaction process.

The liquefaction mechanism for CS with the solvent of PEG400-glycerol was proposed based on the characterization results of the CS, LRs and LP. It is to be noted that the liquefaction process includes the depolymerization reaction and repolymerization reaction. As depicted in Figure 6a, the cellulose and hemicellulose are degraded by cleaving the C–O bond in the presence of sulfuric acid. First, the hydrogen cations attack the oxygen atoms that are present in C–O–C and then the C–O bonds of the glycosides are broken down to form hydroxyl and carbocation. The decomposition of lignin in CS has a similar mechanism by cleaving the dominant linkages, including β–O–4, 4–O–5 and dibenzodioxocin units. Hydrogen cations attack the hydroxyl group of the lignin with the removal of the water molecule. Therefore, the depolymerization reaction of CS could be classified into the mechanism of electrophilic substitution reaction attacked by the hydrogen cation.

Meanwhile, as shown in Figure 6b, the repolymerization of biomass derivatives took place synchronously with the depolymerization of CS in the liquefaction process. In the acidic conditions, the lignin derivatives are attacked by the hydrogen cation to form a cationic intermediate. Then the PEG400 reacts with the intermediate via the nucleophilic substitution to form the new ether bonds and followed by the release of hydrogen cation from the same hydroxyl groups in the PEG chain. Through a similar mechanism, the lignin derivatives repolymerization with the cellulose and hemicellulose derivatives and the self-condensation of lignin derivatives are realized. Therefore, the repolymerization reaction of biomass derivatives could still be classified into the mechanism of electrophilic substitution reaction attacked by the hydrogen cation.

## 4. Conclusions

Corn stalk was successfully liquefied with PEG400 and glycerol as the liquefaction solvents and the sulfuric acid as the catalyst. An optimum liquefaction yield of 95.39% was obtained with the acid content of 20% at 150 °C for 120 min. The characterization results indicated that the depolymerization of CS and the repolymerization of biomass derivatives could take place simultaneously in the liquefaction reaction of corn stalk. Depolymerization of corn stalk mainly occurred at a lower temperature of <150 °C, resulting in abundant constituents with easy pyrolysis characteristics in the LRs. Moreover, repolymerization, including the reaction of lignin derivatives with cellulose derivatives, hemicellulose derivatives and PEG400 and their self-condensation, mainly occurred at the high temperature of 170 °C. Thus, the corresponding liquefaction residue (LR-170) exhibited high thermal stability. Furthermore, the solvolysis liquefaction of CS could be classified into the mechanism of electrophilic substitution reaction attacked by the hydrogen cation. This research inspires us to further investigate the solvolysis liquefaction process of biomass for producing biomass-based chemicals.

## Figures and Tables

**Table 1:** The main weight-loss stage of CS and LRs in the DTG curves.

Samples	T_Max1_ (°C)	T_Max2_ (°C)	T_Max3_ (°C)
CS	210 ± 1	320 ± 1	–
LR-130	265 ± 1	348 ± 1	–
LR-150	270 ± 1	376 ± 1	546 ± 1
LR-170	283 ± 1	392 ± 1	552 ± 1

**Table 2:** Elemental analysis of CS and LRs.

Samples	C (wt %)	H (wt %)	N (wt %)	O ^a^ (wt %)	H/C	O/C	HHV (MJ kg^−1^)
CS	45.74 ± 0.21	6.22 ± 0.08	1.39 ± 0.11	46.65	0.1359	1.02	16.04
LR-130	43.12 ± 0.15	6.16 ± 0.12	0.66 ± 0.03	50.06	0.1428	1.16	14.44
LR-150	37.83 ± 0.30	5.10 ± 0.15	0.92 ± 0.08	56.15	0.1348	1.48	10.03
LR-170	39.15 ± 0.19	4.57 ± 0.10	0.98 ± 0.05	55.30	0.1167	1.41	9.87

^a^ The O is obtained by difference.

## Supplementary Materials

The following are available online at https://www.mdpi.com/2073-4360/12/10/2337/s1, Table S1: GC-MS analysis results of liquefied products from optimum conditions.
